# Physico-Chemical Properties of Clay Minerals and Their Use as a Health Promoting Feed Additive

**DOI:** 10.3390/ani9100714

**Published:** 2019-09-23

**Authors:** Małgorzata Nadziakiewicza, Sylvia Kehoe, Piotr Micek

**Affiliations:** 1 Department of Animal Nutrition and Dietetics, University of Agriculture in Kraków, al. Mickiewicza 24/28, 30-059 Kraków, Poland; malgorzatanadziakiewicz@gmail.com; 2 Department of Animal and Food Science, University of Wisconsin—River Falls, 410 S. 3rd Street, River Falls WI 54022, USA; sylviakehoe@gmail.com

**Keywords:** phyllosilicates, clays, feed decontamination, mycotoxins, animal health, product quality

## Abstract

**Simple Summary:**

Feeds contaminated by mycotoxins cause organ damage, immune suppression and health disorders, limiting growth and performance of farm animals. One of the most practical approaches to minimizing the negative effect of these substances in livestock diets is by the use of different feed additives, preventing their absorption from the gastrointestinal tract. Growing interest in particular is applied to mineral additives, such as clays and clay minerals. These materials are not digested but bind toxic metabolites and mycotoxins, moving them through the digestive tract, without detrimental influence on the animal body. Apart from the binding capacity, clay minerals show other activity which could positively affect animal welfare and productivity. However, each type of clay has its own specific binding capacity that can greatly vary according to its origin deposit and treatment. Moreover, clays may bind with not only noxious substances but also some nutrients, causing a nutritional imbalance for animals. Therefore, the aim of the review was to present the available knowledge on the properties and use of clays in feedstuff decontamination as well as to discuss the resulting potential benefits for animal health and safety of human food.

**Abstract:**

The contamination of feeds with microbiological or toxicological agents can affect health, productivity and safety of livestock animals and their products. The treatment of feedstuffs to lower the content of undesired substances before feeding is expensive and labor intensive, therefore an alternative is to reduce their gastrointestinal absorption. Different feed additives are available, however the use of clays and clay minerals are ideal for this purpose due to their high specific surface area, adsorption capacity, low or null toxicity for the animal and low cost. A large number of clays available to producers have different structures that are dependent on their mining source, causing difficulty in proper categorization. For this reason properties of phyllosilicates with 1:1 layers (one sheet of SiO_4_ tetrahedra joined to one sheet of Al- or Mg-octahedra), 2:1 layers (one sheet of Al- or Mg-octahedra between two sheets of Si-tetrahedra), and 2:1:1 layers (a basic 2:1 structure with an interlayer brucite (with cations Mg^2+^ or Fe^2+^) or gibbsite (with cation Al^3+^) sheet) and tectosilicates are described. The role of clay minerals in animal production shows a reduction in diarrhea, better feed conversion ratio, and improved health of many livestock species due to their specific adsorption potential of many feed mycotoxins. Overall, there is growing interest in the use of clays due to their beneficial characteristics, absence of primary toxicity and success in research to reduce animal disease and improve animal production and safety of animal products.

## 1. Introduction

Feed contamination with microbiological or toxicological agents can affect livestock health, reproduction and productivity and, at the same time, may reduce the safety of animal-derived food products such as milk, meat and eggs. In this context, feed quality is the most crucial factor that can lead to exposing animals to contaminants. Different technical processes such as cleaning, disinfection or heating are employed to damage or destroy undesirable substances or pathogens. Several practices have been especially developed for the prevention or reduction of mycotoxins contamination [1].

Treatments resulting in reductions of fungal and mycotoxin contamination of feedstuffs before feeding are often not feasible since they are labor and cost-intensive. For this reason, the most practical approaches to minimizing the negative effect of mycotoxins in livestock diets is by the use of different feed additives, commonly called mycotoxin adsorbents or binders, preventing their absorption from the gastrointestinal tract [1,2]. Classification and evaluation of detoxification agents is complex. The limitations associated with organic adsorbents, such as different extractions from some fiber-rich plants (oat, lucerne) or the yeast cell wall, point to the use of inorganic absorbents due to their practicality (lower costs, higher operability and less sensitivity to varying gastrointestinal conditions). One example are mineral additives, such as aluminosilicates, which are not digested but bind toxic metabolites and mycotoxins, moving through the digestive tract in the unchanged form, usually without any detrimental influence on the animal body [2]. The use of clays and clay minerals in the feed industry as a contaminants binder is caused by their physical and physico-chemical characteristics [3]. The fundamental properties for which clay minerals are used in animal nutrition are their high specific surface area, adsorption capacity, cation exchange capacity (CEC), thixotropy and colloidal properties, favorable rheological characteristics, swelling capacity, dispersivity, chemical inertness, low or null toxicity for the animal, and low cost [4].

Clays have unique properties that are characteristic for their particular place of origin. Therefore, with regards to the wide range of clay sources and their possible application, the aim of the review was to present the available knowledge on the properties and use of clays in feedstuff decontamination as well as to discuss the resulting potential benefits for animal health and safety of human food.

## 2. Historical Focus

The beneficial effect of the use of clay minerals for animals was noted a long time ago. In the second century A.D., Galen, the Greek philosopher and physician, was the first to record the use of clay for sick or injured animals [5]. Currently, the future of the animal production will evolve based on a rapidly growing global population and the constant pursuit for greater efficiency. For this reason, in animal nutrition, numerous natural substances have been scientifically tested [6]. Clay minerals are in the center of this interest. The natural zeolites, particularly clinoptilolite (CPL), are widely used by livestock farmers as feed additives for beef cattle, dairy cows, sheep, goats, pigs and poultry due to their detoxifying/decontaminating properties and abilities to act as reducers of heavy metals, organic pollutants, radionuclides and antibiotics. The CPL was even tested as a feed supplement for the treatment of infected honeybee colonies [7].

Nowadays, According to Regulation (EC) No 1831/2003 of the European Parliament and of the European Council concerning additives used in animal nutrition, all clay material additives belong to the category ‘technological additives’. They are represented in functional groups by: binders, substances for control of radionuclide contamination and anticaking agents. The European Commission has registered 15 clay minerals allowed for use as additives in animal nutrition but some of them have no assigned functional group.

## 3. Mineral Structure of Clays

**Figure 1:**
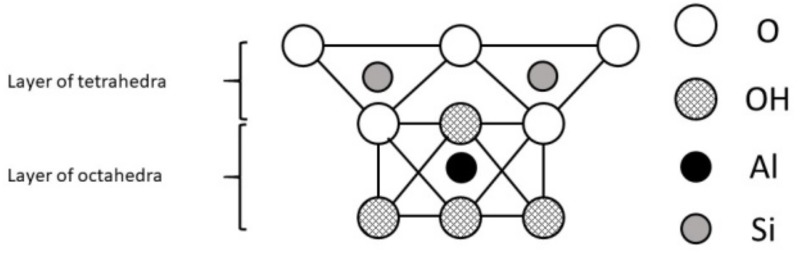
The 1:1 layer phyllosilicate structure.

**Figure 2:**
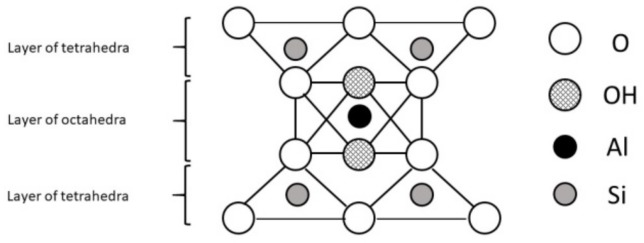
The 2:1 layer phyllosilicate structure.

A mineral is an element or chemical compound that is normally crystalline and has been formed as a result of geological processes [4]. They were formed under specific conditions which influenced their structure and properties. Aluminosilicates, minerals composed of aluminium, silicon, and oxygen, plus counteractions constitute the most abundant group of rock-forming minerals including clays. The basic structural unit of silicate clay minerals consists of the combination of silica tetrahedral and aluminium octahedral sheets, both with oxygen and hydroxyl groups [8]. However, the definitions of clay minerals still are not unanimous, because they may contain different associated minerals and impurities such as quartz, cristobalite, alunite, iron oxides, anatase, magnesite, serpentine and others [9]. Primary materials are formed at elevated temperatures and pressures, and are usually derived from igneous or metamorphic rocks. Some of them formed secondary minerals of more stable structure. These secondary minerals are often referred to as phyllosilicates because they exhibit a platy or flaky nature (Greek: phyllon-leaf [10]). Differences in structures of clay minerals give them different physical (especially the distribution of negative and positive charges on surfaces and type of bonds between atoms and molecules) and chemical properties (i.e. associated atoms, type of ions and their exchangeability). This, as well as distance between layers, affects their use as feed additives and expected results. Two types of phyllosilicates are distinguished: 1:1 layer type (T-O; Figure 1) consist of one sheet of SiO_4_ tetrahedra joined to one sheet of Al- or Mg-octahedra and 2:1 (T-O-T; Figure 2) layer type consist of one sheet of Al- or Mg- octahedra between two sheets of Si-tetrahedra [4].

The 1:1 layer minerals are represented by a kaolinit group while 2:1 layer minerals are represented by 3 subgroups: Smectite, mica, and talc. They have different properties which can influence their application. Smectites have exchangeable cations located between water molecules in the interlayer. When the mineral is saturated with water, the basal spacing between layers can increase, while under dry conditions, the basal spacing may be reduced. This trait found in smectites is often referred to as shrink-swell potential. Contrastingly, the strong bonding of the interlayer cations in vermiculites holds the 2:1 layers together, limiting expansion of the basal spacing [10].

**Figure 3:**
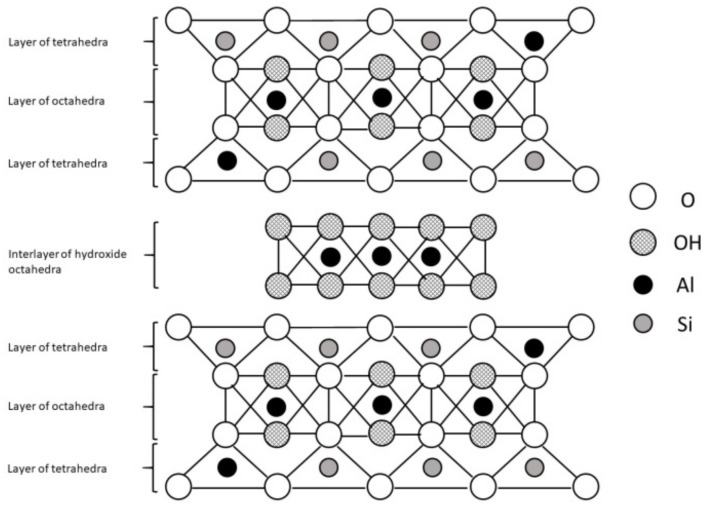
The 2:1:1 layer phyllosilicate structure.

There is also an additional group of phyllosilicates, the 2:1:1 layer type, which have a basic 2:1 structure with an interlayer brucite (with cations Mg^2+^ or Fe^2+^) or gibbsite (with cation Al^3+^; Figure 3) sheet. This group is represented by chlorites. Since there is no water adsorption within the interlayer space, they are considered as nonexpansive minerals [11].

## 4. Aspects Influencing Phyllosilicate Properties

Phyllosilicates (one of the major groups of aluminosilicates) are a class of rock-forming minerals with porous sheet structure with different distances between layers. The electrical conductivity (mS/m) of a porous material is the combination of electrical conductivities of the matrix material and the pore fluid [12]. A number of possible charged ions by the negatively charged surface of clay materials, called cation exchange capacity (CEC), depends not only on the number of sheets but also on the cations located in these structures [13]. Therefore, chemical properties of clay minerals depend significantly on their structure, their cation- and anion-exchange, and adsorption ability which is what determines their roles in different applications.

### 4.1. Properties of Phyllosilicates with 1:1 Layers

The phyllosilicates with 1:1 layers that are used as feed additives are represented by the kaolinit group of minerals.

#### Kaolinit Group-Kaolin and Halloysite

Kaolin is a clay that contains 10–95% (usually 85–95%) of the mineral kaolinite [10]. The cation exchange capacity of kaolinite is considerably less than that of montmorillonite (MMN, smectite group), depending on the particle size, but the rate of the exchange reaction is rapid, almost instantaneous. The adsorbed material can be easily removed from the particles because adsorption is limited to the surface of the particles (planes, edges), unlike the case with MMN, where the adsorbed molecules are also bound between the layers [14].

Halloysite usually contains a minor amount of metal ions replacing aluminum in some positions. Halloysite consists of nanotubes (HNTs) which form in multiple rolled layers composed by a sheet of corner-sharing SiO_4_ tetrahedral bonded of edge-sharing AlO_6_ octahedral [10]. Between the aluminosilicate layers, there are crystallographic water molecules and kaolinite OH groups. Halloysite occurs mainly in two polymorphs, the hydrated form with interlayer spacing of 10 Å and the anhydrous form with interlayer spacing of 7 Å. This structure has negative charges on its external surface, positive charges on its inner lumen surface, and negative/positive charges at its edges. The hydroxyl groups at the surfaces and edges of HNTs provide a useful opportunity for modification with various organic compounds. The length of nanotubes depend on the deposit and purification procedure which influence its properties [15].

### 4.2. Properties of Phyllosilicates with 2:1 Layers

The phyllosilicates with 2:1 layers, used as feed additives, belong to 3 groups of minerals: smectite, mica and talc.

#### 4.2.1. Smectite Group-Bentonite (Montmorillonite)

Bentonite is a heterogeneous rock formed of highly colloidal and plastic clays composed mainly of MMN [16]. Minerals of the smectite group have exchangeable cations, which can be easily substituted with other cations which creates absorption capability on sheet surfaces. These cations can be replaced reversibly. There are two types of bentonite: sodium bentonite, which has a single water layer containing Na^+^ as exchangeable ions (with swelling properties) and calcium bentonite with a double water layer containing Ca^2+^ exchangeable ions (a non-swelling mineral). Bentonite has the ability to absorb large quantities of water with an accompanying increase in volume (12–15 times) and can form thixotrophic gels. Montmorillonite, the main component of bentonite, has a high cation exchange capacity, which is little affected by particle size [17].

#### 4.2.2. Mica Group-illite and Vermiculite

Illite has a structure like MMN but its layers are bonded together by potassium ions. Nieto et al. refer to the fact that the term illite is used in clay mineral literature in most cases without a strict mineralogical meaning but usually meaning dioctahedral mica [18]. The crystal structure of illite is poorly known, due to its small grain size and defective character. The pattern and composition of illite are similar to muscovite and other micas with an interlayer cation deficit. Illite’s balancing cation is mainly potassium, which is not easily exchangeable.

Vermiculites are mostly trioctahedral with Mg^2+^ as the dominant octahedral cation [19]. Between layers are hydrated exchangeable cations. The interlayer material takes the form of an incomplete octahedral sheet, similar to the interlayer sheet in chlorite. The flakes expand forming “worm-like” macrostructures but during heating, the loss of interlayer water molecules cause a collapse of the structure. Exceeding specified temperatures may cause irreversible changes in vermiculite structure [20].

#### 4.2.3. Talc Group-Steatite

Steatite, also known as soapstone or soaprock, is a type of metamorphic rock in which talc (hydrous magnesium silicate) is mixed with minerals such as chlorite, amphibole (inosilicate group) and carbonates. Soapstones were formed from volcanic tuff and basalt and have different properties due to varying mineral content. Some soapstones are massive, but most are schistose and sometimes full of fissures. If the talc content is very high, approaching 100%, the term steatite is often used [21]. Steatite is relatively soft because of its high talc content and can vary by its different colors and grades. Talc may contain other minerals as impurities: quartz, calcite (calcite group), dolomite (dolomite group), magnesite (calcite group), serpentine (kaolinite group), chlorite (chlorite group), tremolite (inosilicate group) and anthophyllite (inosilicate group) [22]. Low moisture content, absorption ability, chemical inertness, and good retention made these minerals useful as feed additives.

### 4.3. Phyllosilicates with 2:1 Layers with Joined Strips

The phyllosilicates with 2:1 layers with joined strips used as feed additives belong to the palygorskite-sepiolite group of minerals.

#### Palygorskite-Sepiolite Group-Palygorskite (Synonym Attapulgite) and Sepiolite

The palygorskite and sepilolite are structurally similar with different arrangements of the silica and alumina sheet structures [23]. These phyllosilicates contain a continuous two-dimensional tetrahedral sheet and discontinuous octahedral sheets. They have a ribbon structure, where each of the ribbons are connected to the next by an inversion of SiO_4_ tetrahedra along a set of Si-O-Si bonds. These clays usually contain impurities of quartz, feldspars, carbonates, gypsum, cristobalite, smectite, illite, kaolinite, chlorite, and iron oxides. Micropores, channels and fine particle size, together with fibrous structure, create a high surface area. Palygorskite occurs as bundles of agglomerate needle-like structures which disperse in water and other polar solvents yielding a randomly intermeshed network of fibers in the solvent [24].

Sepiolite is a fibrous clay mineral that differs from laminar clays by having tunnels in its structure. These tunnels can hold water as well as other small molecules. The physicochemical properties of sepiolite depend on the crystalline structure at an atomic scale. The tunnels in the crystal cell induce a fibrous morphology which is responsible for the great absorptive power and the good rheological properties of this mineral [25]. In the nature sepiolite is usually associated with bentonite and both have similar properties such as high surface area and significant sorption capacity. However, the cation exchange capacity of sepiolite is much lower than that of smectite [8].

### 4.4. Phyllosilicates with 2:1:1 Layers with an Interlayer of Brucite or Gibbsite Sheet

This group is represented by chlorites.

#### Chlorite Group-Chlorite

Chlorite is a 2:1:1 layer type silicate formed under a wide range of conditions. It has T-O-T (tetrahedral-octahedral-tetrahedral) structure interlayered by additional hydroxide octahedral sheets. Chlorite minerals have a 2:1 layer structure with an excess of negative charge. The excess charge is balanced by a positively charged interlayer hydroxide sheet [4]. Low CEC of chlorites can influence the application of this mineral.

## 5. Properties of Tectosilicates

Different from the phyllosilicates class of rock-forming minerals are tectosilicates, also called polysilicates.

### 5.1. Zeolite Group-Zeolite

Zeolites are crystalline-aluminosilicates, composed of SiO_4_ and AlO_4_ tetrahedra joined into 3-dimensional frameworks with molecular dimensional pores which form larger molecular sieves. A negative framework charge caused by the presence of aluminum is balanced by an exact number of cations required to make it neutral. These cations are relatively mobile, and can be replaced by other cations [26]. Zeolites have a honeycomb-like structure with microspores which gives them the ability to easily accommodate simple organic molecules. The combination of cation-exchange and microporous capacity confers particular properties to zeolites what allows for many applications [27]. Among numerous types of natural zeolites, CPL which incorporates a biologically active nanoporous structure is the most widespread and scientifically studied zeolite [7].

### 5.2. Zeolite Group-Natrolite-Phonolite

Natrolite-phonolite is a natural mixture of aluminum silicates, natrolite, and feldspar (tectosilicate Al-Si framework group). Natrolite belongs to the zeolite family. Feldspar subgroup minerals are among the most common rock-forming minerals of planetary crusts. On Earth, they occur in many type of igneous, metamorphic and sedimentary rocks [28]. There are 4 chemical groups of feldspar: potassium, sodium, calcium and, less frequently, barium.

## 6. Reduction of Mycotoxin Contamination of Feeds

The problem of contamination of animal feeds with mycotoxins has been an area of great interest since 1960, when the cause of the massive death of ducklings in the United Kingdom had been established [29]. Mycotoxins are toxic metabolites produced by several fungi species. There is an increasing concern of the effects of mycotoxins on animal health and wellbeing [2]. Even low concentrations of these substances in the long term can be damaging. Aflatoxin (AF), deoxynivalenol (DON), zearalenone (ZEA), fumonisin (FUM), and ochratoxin (OTA) are the five major mycotoxins that are commonly found in grains used as feedstuffs around the world. They can develop during growth, harvesting, drying or storage of fruits, seeds, grains and by-products that are widely used in food and feed preparations [8,30]. The mycotoxins commonly occurring in cereal grains are not destroyed during most technological operations [1]. Usually food processing affects mycotoxin distribution and concentrates mycotoxins into by-products commonly used as animal feeds [31]. In turn, they can become residues in animal products and enter the human food supply chain. Even in the fermentation process, the content of mycotoxins may increase. After brewing processes, Pinotti et al. found significant concentration of ZEA, DON, OTA, FUM, and AF in beer [32].

Feeds contaminated by mycotoxins can decrease growth and productivity, cause organ damage, immune suppression and be carcinogenic, mutagenic, teratogenic and estrogenic for animals. Therefore, mycotoxin contamination greatly affects the health and economic stability of many farm industries [30]. The EU Commission recommends increased monitoring for the presence of DON, ZEA, OTA and FUM in cereals and cereal products intended for animal feeding (Regulation EU 2017/625 of the European Parliament and of the Council). The main method for protecting animals against mycotoxicosis is the addition of feed adsorbents to bind mycotoxins efficiently in the gastro-intestinal tract. Physical decontamination processes are favored [2] because using a chemical decontamination process is not legal within the EU (Directive, 2002/32). In this regard, aluminosilicates are the preferred adsorbents, followed by activated charcoal or special polymers [33].

Using clay minerals for mycotoxin binding involves physical and chemical adsorption. This features relies on basic chemistry principles: hydrophobic bonding (complex process involving more than one type of bond), hydrogen bonding, electrostatic interaction of attraction or repulsion, Van der Waals interactions, coordination bonds, and cation exchange. Other physical characteristics, like specific surfaces, can also play a role in the binding capacity of clay mycotoxin binders [8]. Different types of interactions often occur simultaneously. Each type of mineral binder has its own specific binding capacity that can vary a lot according to its origin deposit and treatment (chemical, physical or thermic). Even clays from the same family but from different deposits can have different efficiencies against these substances [34]. Some adsorbents can be used to minimize the toxic effects of various mycotoxins while others are able to adsorb only specific ones [33]. However, clays may bind not only noxious substances but also some nutrients like vitamins and trace elements, causing a nutritional imbalance for animals [3,27,35].

### 6.1. Aflatoxin (AF)

Contamination of food with AF is a global problem. Aflataoxins are secondary metabolites produced by the fungi *Aspergillus flavus* and *A. parasiticus* and are immunosuppressive, anti-nutritional, and mutagenic. Feed contamination with AF may occur during pre- or postharvest contamination of crops [36]. Among 18 different types of AF produced by *A. flavus* strains, AF B1 is the most toxic, mutagenic and is classified by the World Health Organization and the International Agency for Research on Cancer as a group 1 carcinogen. Aflatoxins can occur in milk and in dairy products made from milk as a result of feeding contaminated rations to dairy cattle [37]. A very promising strategy used to mitigate exposure is the inclusion of high affinity AF adsorbents in the diet [38,39]. Many research trials are conducted to find satisfactory mycotoxin binders in order to prevent animal toxicity. For this purpose, the properties of various clay minerals have been tested and described in many publications [33,36].

Aflatoxins are dangerous for animal health, create economic losses and may be dangerous for humans after transferring to food of animal origin. In this context Ca-MMN fed to cows help to prevent AF residues in milk [33]. The addition of vermiculite, nontronite (smectite group), and MMN to the diet reduced AF transfer from the rumen to the milk [40]. Schoonmaker reported that smectite clays may be used as adsorbents of mycotoxins in a normal physiological range of ruminal pH [41]. The addition of smectite clays to dairy cattle diets reduced concentrations of AF M1 (4-hydroxylated metabolite of AF B1) by 40 to 48% in milk. Montmorillonite is an adsorbent with a high affinity for AF and can be used for the preventive management of aflatoxicosis in pigs [42].

Physical and chemical structures of smectite clays let them absorb mycotoxins, tannins, heavy metals, bacteria, and viruses and expel them from the body. Bentonite is a common smectite clay mineral fed to livestock for this purpose. Zeolite has similar properties but cage-like structures and tube forms within its molecular structure can incorporate a variety of molecules and ions [41]. In vitro, isotherm studies by Di Gregorio et al. showed that in a gastro-intestinal tract model, the following clays had the capability to bind AF: bentonite, diatomite (siliceous-rock), hydrated sodium calcium aluminosilicate (HSCAS, zeolite group), MMN and zeolite [8].

### 6.2. Ochratoxin (OTA)

Ochratoxin can be produced by a variety of mold fungus species including: *Penicillium verrucosum, P. nordicum, Aspergillus carbonarius, A. niger, A. westerdijkiae, A. steynii* and *A. ochraceus*. It is found in a large variety of agricultural commodities and is recognized as a potential health risk, mainly toward humans. In farm animals, the intake of feed contaminated with OTA affects animal health and productivity, and may result in the presence of OTA in the animal products [37]. Therefore OTA has been classified as a possible human carcinogen (group 2B) by the International Agency for Research on Cancer [38]. According to Di Gregorio et al., the clays with a capacity to bind OTA were: diatomite and HSCAS [8].

### 6.3. Zearalenone (ZEA)

Zearalenone is a mycotoxin produced by several species of the genus *Fusarium*, including *F. acuminatum*, *F. crookwellense*, *F. culmorum*, *F. equiseti*, *F. graminearum*, *F. oxysporum*, *F. semitectum* and *F. sporotrichioides*. Zearalenone more commonly occurs in the field, but can also be a postharvest mycotoxin. Swine are the most affected animals by ZEA, although poultry, cattle, and rodents can also show signs of toxicity after ingesting contaminated grains [30]. Zearalenone has important estrogen-like activity, which leads to deficiencies in conception, ovulation, embryo implantation in animals, induced abortion and other problems like adverse liver lesions. Exposure on ZEA is associated with carcinogenic, genotoxic, and immunotoxic effects in animals and humans [43]. Abbes et al. classified HSCAS and a phyllosilicate clay of the smectite class (belonging to the MMN group) as effective in protection of ZEA-induced cell damage [44]. In in vitro studies clays capable to ZEA binding were bentonite, diatomite, HSCAS, MMN and zeolite [8]. De Mil et al. screened 27 binders, commercially available in Belgium and The Netherlands for their ZEA adsorption ability [45]. In this regard they showed an effectiveness of zeolite, sepiolite, CPL, MMN, calcite, dolomite, mica, kaolin, quartz or their mixtures. Similarly Wongtangtintan et al. checked adsorption effectiveness of the clays in order to prevent ZEA induced disease of animals [46]. They found that bentonite was more effective adsorbing ZEA than acid-activated MMN, CPL or Ca-montmorillonite (Ca-MMN).

### 6.4. Fumonisin (FUM)

Fumonisin are produced by some fungi of genus *Fusarium*, almost all strains of *F. verticillioides* syn. *Moniliforme*, and many strains of *F. proliferatum*. It can be found mainly in maize but also in other grains. Contamination of FUM can cause immunologic effects, hepatotoxic effects and nephrotoxicity. It is classified as a carcinogen [37]. There are some potential health effects associated with FUM feed contamination such as liver and kidney disorders, possible indirect mutagenicity (DNA damage), and negative effects on reproductive performance. Fumonisin has been shown to have the potential to cause cancer which is thought to arise following disruption of fat metabolism by the toxin’s immunotoxicity. Birth defects caused by the potential neurotoxicity of FUM, leukoencephalomalacia (softening of brain tissue) in horses, pulmonary edema in pigs, and nephrotoxic effect in horses, pigs, sheep, rats, mice, and rabbits have also been reported [47,48]. Baglieri et al. tested natural sodium MMN (Na-MMN) in which the inorganic cation had been exchanged with an ammonium organic cation [49]. The modified clays showed to be more efficient than natural ones as FUM absorbents. In turn, Jaynes and Zartman indicated that different approaches are needed for the different properties of AF and FUM to limit toxicity in animal feeds [50]. They described an animal adsorption study which showed that FUM is a very water-soluble mycotoxin and, unlike AF, FUM adsorption to feed additives is pH-dependent. Fumonisins adsorb effectively to MMN by ion exchange.

### 6.5. Deoxynivalenol (DON)

Deoxynivalenol, also known as vomitoxin, belongs to a large class of mycotoxins called trichothecenes and is a naturally-occurring mycotoxin produced by many strains of *F. graminearum* and *F. culmorum* [38]. DON is one of the most prevalent mycotoxins in temperate regions of the world. It commonly contaminates corn, wheat, oats, and barley, which can be co-contaminated with other trichothecenes and ZEA produced by strains of *Fusarium* under similar environmental conditions. Deoxynivalenol can occur also after harvest if grains are stored under high moisture conditions [30] and was discovered in wheat based foods such as flour, bread, and baby foods. The trichothecenes are strong inhibitors of protein biosynthesis. It can cause animal disease and stimulate feed refusal, vomiting, immunosuppression, and loss of productivity. Swine are more sensitive to DON than poultry or cattle [37]. Clay minerals are considered to be effective DON binders. *In vitro* gastro-intestinal tract model showed that clays capable of binding to DON were: bentonite, diatomite, sepiolite and zeolite [8]. Qiang et al. reported zeolites and bentonites as adsorbents prevented DON, FUM B1 and ZEA toxicity and decreased animal health disorders [51]. The potential adsorption of mineral binders (smectite, quartz, dolomite, feldspar, mica, kaolinite, and illite) to DON and ZEA were also shown in vitro by Sabater-Vilar et al. [52].

## 7. Health and Performance of Animals Fed Clay Minerals

Apart from the mycotoxin binding capacity, clay minerals show other activity which could positively affect animal welfare and productivity. Reducing the volume of harmful gastro-intestinal gases, the number of pathogens or the speed of food passage through the intestinal tract allows domestic animals to carry out more efficient digestion of proteins and other nutrients [3]. Clays reduce the speed of passage of feed along the digestive tract which allows more time for digestion. Feeding clays also causes morphological changes in the intestinal mucosa such as an increase in villus height and an increase in the villus height to crypt depth ratio. These changes increase the surface area of the gastrointestinal tract thus increasing nutrient digestibility [35]. Likewise Zhou et al. [53] have shown that supplementation with zeolite affects digestive enzyme activities in jejunal digesta and mucosa. It increased lipase, trypsin, maltase and sucrase activity and enhanced the digestibility of nutrients. This helps increase animal growth and improve meat and derived final product quality. In addition to achieving better growth parameters, Al-Beitawi et al. [54] described positive effects on blood biochemical profiles of broiler chicken when fed nanoclay minerals. Their research significantly increased total protein, albumen, globulin and high-density lipoprotein (HDL) concentrations and lowered triglycerides and low-density lipoprotein (LDL) concentrations.

In this context, clay minerals could become suitable feed additives that ensure good health and growth of animals. Actually, some changes in the structure of clay minerals make it possible to search for applications in the protection of farm animals against pathogenic bacteria [3]. Xia et al. describe that Ca^2+^ ion replaced by [Cu (AlO)_n_(H_2_O)_4-n_]^x+^ or Cu^2+^ in MMN caused a change of electrical balance to a surplus which allowed them to accumulate *Escherichia coli* and *Salmonella enteritidis*, whose cell walls have a negative charge [55]. In this context, an in vitro study demonstrated that MMN could adsorb *E. coli* as well as treat intestinal infections caused by *Salmonella* [56]. In addition, MMN could mitigate the mycotoxin-induced adverse effects on poultry growth performance, oxidation status, and immune functions [57]. In turn, Rodriguez-Rojas, showed that sepiolite is being widely used as a feed additive supplied to broiler chickens and pigs [58]. Uriyanghai pointed to the important role of grits in the digestion of hard ingredients from poultry and examined whether the addition of zeolite as grits could provide a beneficial indication in broiler nutrition [59]. He concluded the lack of negative impact of zeolite addition on bird weight gain, feed intake, feed utilization, gizzard size, gizzard pH but also underlined no significant beneficial results. The summarized effect of different clay minerals in broiler chickens on total body weight gain (BWG) and the feed conversion ratio (FCR) is shown in Table 1.

Qu et al. proved that Ca-MMN can be used successfully for laying hens as a dietary supplement [68]. A ten week experiment showed a favorable decrease in the feed conversion ratio (FCR) with an increase in egg production, egg mass and shell strength with 0.09% addition of Ca-MMN. Lower concentrations of Ca-MMN in the concentrate mixture (i.e., 0.03% and 0.06%) did not have significant effects on FCR, egg production and egg mass. In contrast, increasing Ca-MMN concentration improved yolk index and shell thickness, and partially enhanced hens’ antioxidant capability and immune function.

Subramaniam and Kim mentioned different clay minerals used as dietary supplements for pigs: smectites, kaolin minerals (kaolinite, dickite, nacrite, halloysite) and zeolites [35]. They indicated numerous benefits such as improving FCR and reducing severity and duration of diarrhea in pigs. This is likely due to increases in numbers of *Bifidobacteria* and *Lactobacillus* and decreases in *Clostridia* and *E. coli* in the intestine of pigs.

Jiao et al. conducted a 14 day experiment feeding MMN with Zn as an additive to a feed concentrate mixture for weaned pigs [69]. They showed higher growth performance of animals in the experimental group in combination with a lower FCR. A decrease in the number of bacteria in the jejunum and colon was also observed. Other research has shown kaolin as an additive that can be used to prevent economic losses caused by diarrhea in piglets during the periods after birth and after weaning and to protect other livestock from diarrheal diseases [3,70]. However, some research shows no improvement of clay mineral additives on animal health or performance. According to Subramaniam and Kim, the effects of clay supplementation on pig performance have been inconsistent and there are many studies which have noted no improvements in pig performance as a result of feeding clay [35]. Younger pigs respond more to dietary clay supplementation than older ones [70], but the final response is most likely affected by the level of supplementation. Usually addition of 1 to 3% of clay in the concentrate mixture for swine is recommended [70,71].

Humer et al. indicated beneficial effects of clay mineral-based supplementation on cattle liver function, even during stress conditions of subacute ruminal acidosis [72]. Acidosis is a serious metabolic disease of cows as a result of consuming too many easily fermentable carbohydrates. Even without clinical symptoms, this metabolic disorder is dangerous and can have many detrimental effects. Lean [73] and Sulzburger [40] showed, that using clay minerals (Na-MMN and dietary clay EcoMix^®^ (United Minerals Group, Kiev, Ukraine) respectively) as an additive to cereal grains for lactating cows is efficient in buffering rumen pH and reducing the time spent below rumen pH 5.6 after grain feeding. In their research, linear treatment effects on rumen and fecal pH showed an increase in pH for increasing clay percentages in the diet. Cows fed clay tended to have higher milk yield and higher 3.5% fat corrected milk (FCM), which confirmed that clays could alleviate symptoms of gastrointestinal stress caused by changes in pH levels. Also, Valpotic et al. concluded that zeolite CPL used as a feed additive improved health and economical parameters in cattle production [7].

## 8. Side Effect of Clay Minerals

Clay minerals registered by the EU as binders are widely used in feed industry to improve pellet quality [74]. Yalcin et al. indicated that sepiolite addition to the feed of dairy cows and fattened cattle resulted in production benefits, decreased energy consumption during pelleting, enhanced the pellet durability index and minimized formation of fine particles [75,76]. Also, bentonite used for aquafeed production improved pellet physical qualities, growth performance of fish, FCR, specific growth rate, and increased red blood cells and hematocrit [77].

Gilani et al. reported the benefits of improving the quality of concentrate mixtures by using bentonites, zeolites or kaolins as pellet binders [74]. In concentrate mixtures for turkeys, sodium bentonite increased the durability of pellets and reduced the moisture and nitrogen content of broiler litter while CPL lowered litter ammonia-nitrogen levels. In turn, zeolite decreased NH_3_ and CH_4_ from laying henhouses. However, achieving the goal of improving the quality of pellets does not always bring a beneficial economic effect [78].

## 9. Conclusions and Future Perspectives

Clay minerals are at the center of attention as feed additives with many beneficial effects. Their natural origin is an important asset. It seems that the growing interest in the negative effects of mycotoxins on animals and humans in connection with the promising results of current research will be a stimulus for their wider use. In this respect, clay minerals are of particular interest due to their specific adsorption properties significantly contributing to animal health. Clays bind noxious compounds and expel them from the body of animals providing production of safe food for human consumption. In addition, other beneficial effects of feeding clays have been observed connected to their antimicrobial properties, alleviation of gastrointestinal conditions by detoxification of anti-nutritional compounds in the feed, preventing diarrheal infections and showing valuable properties as binding agents in the production of pellets.

Clays are useful in current animal feed practice, however still less than ideal. When added into feed at high concentrations, they may decrease the total nutritive value of feed and bind other nutrients like vitamins and trace elements, causing a nutritional imbalance for animals. Therefore, the use of clay minerals as feed additives is not the only practical solution to feed decontamination. Certainly this issue should be adapted to the current demands of farmers and further studied to guarantee the effectiveness and safety of clays as feed additives.

## Figures and Tables

**Table 1:** The effect of clay mineral additive on total body weight gain (BWG) and feed conversion ratio (FCR) changes of broiler chickens.

References	Clay Mineral	Duration of Experiment (day)	Amount of Clay (%)	Changes in Relation to Control (%)	
				BWG	FCR
Safaei Kotouli et al. [60]	Kaolin	42	1.5	5.4	−3.1
			3.0	6.0	−7.2
	Bentonit	42	1.5	0.4	−12.9
			3.0	5.3	−1.1
	Zeolit	42	1.5	6.3	−1.5
			3.0	5.3	−5.2
Eser et al. [61]	Sepiolite	42	0.5	2.1	−2.2
			1.0	2.6	−2.8
Khanedar et al. [62]	Sodium bentonite	42	1.0	3.0	−7.6
			1.5	−0.8	1.5
	Calcium bentonite	42	1.0	2.0	−7.0
			1.5	−0.3	2.0
Owen et al. [63]	Kaolin	56	1.0	−9.0	−4.0
			2.0	−9.5	3.3
			3.0	1.3	−5.0
Nikolakakis et al. [64]	Zeolite	42	1.5	7.2	−1.2
			2.0	10.9	−4.2
			3.0	10.6	−4.3
Owen et al. [65]	Kaolin	56	1.0	−3.3	−5.0
			2.0	−7.4	2.9
			3.0	−0.9	−5.4
Safaei Kotouli et al. [66]	Kaolin	42	1.5	−	−3.1
			3.0	−	−6.4
	Bentonit	42	1.5	−	−0.5
			3.0	−	1.0
	Zeolit	42	1.5	−	−0.5
			3.0	−	−4.2
Zhou et al. [53]	50% Zeolite + 50% Attapulgite	42	2.0 (exp. I)	1.9	2.2
			2.0 (exp. II)	8.5	−1.1
Yalcin et al. [67]	Sepiolite	42	1.0	3.3	−4.9
			2.0	−1.4	−1.8
